# Recurrence of Primary Vascular Leiomyosarcoma Five Years after Initial Diagnosis in the Lower Extremity

**DOI:** 10.1155/2018/3094616

**Published:** 2018-06-05

**Authors:** Hilal Erinanc, Mehmet Ozulku, Aysen Terzi

**Affiliations:** ^1^Konya Uygulama ve Arastırma Hastanesi, Pathology Department, Medicine Faculty, Baskent University, Selcuklu, Konya, Turkey; ^2^Konya Uygulama ve Arastırma Hastanesi, Cardiothoracic Surgery Department, Medicine Faculty, Baskent University, Selcuklu, Konya, Turkey; ^3^Pathology Department, Medicine Faculty, Baskent University, Ankara, Turkey

## Abstract

Primary leiomyosarcomas of vascular origin are rare tumors. They frequently arise within the inferior vena cava; however, the peripheral vein was also affected. To date, only a few hundred cases have been reported in the world literature. Although it is an extremely aggressive tumor, the symptoms may be unspecific, especially in the lower extremities. In this report, we present a case of primary vascular leiomyosarcoma, arising from the short saphenous vein, with symptoms mimicking thrombus in the initial diagnosis. The diagnosis of leiomyosarcomas was confirmed by standard H&E staining and immunohistochemical staining. Recurrence of the tumor has been observed five years after surgical treatment. Due to its rarity, experience in the management of this type of tumor is limited. The mainstay of treatment for these tumors is complete surgical resection. The purpose of the presented case is to discuss the clinicopathological features and management options of this tumor, under the light of the most recent literatures.

## 1. Introduction

Primary tumors arising from the vessel wall are rare. Although primary vascular leiomyosarcoma (vLMS) arising from larger blood vessels (particularly in the inferior vena cava and large veins of the lower extremity) has been reported [1], leiomyosarcoma originating from a peripheral vein is exceptional. A case of primary vLMS of the short saphenous vein has been described in 1998 [2]. Herein, we report a vLMS primarily localized within the wall of the short saphenous vein, which is, to the best of our knowledge, the second presented in the medical literature.

## 2. Report of a Patient

**Figure 1:**
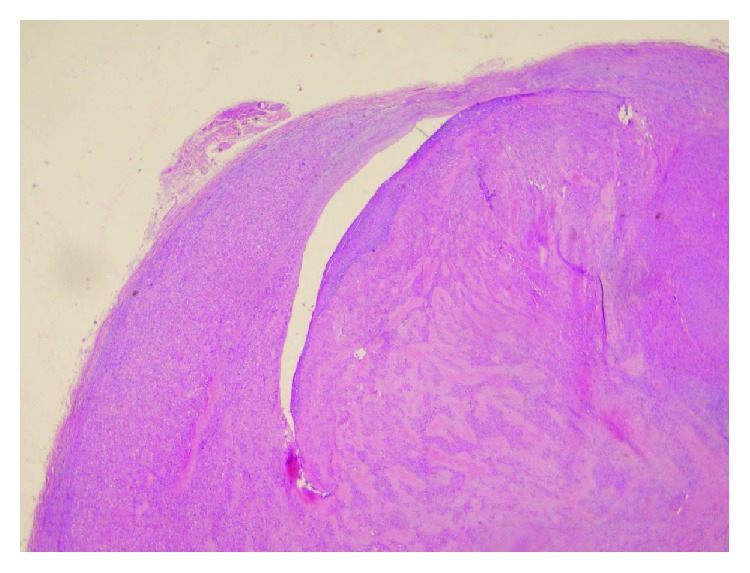
Tumor was arising from the vascular wall and protrude to lumen H&E 10x.

**Figure 2:**
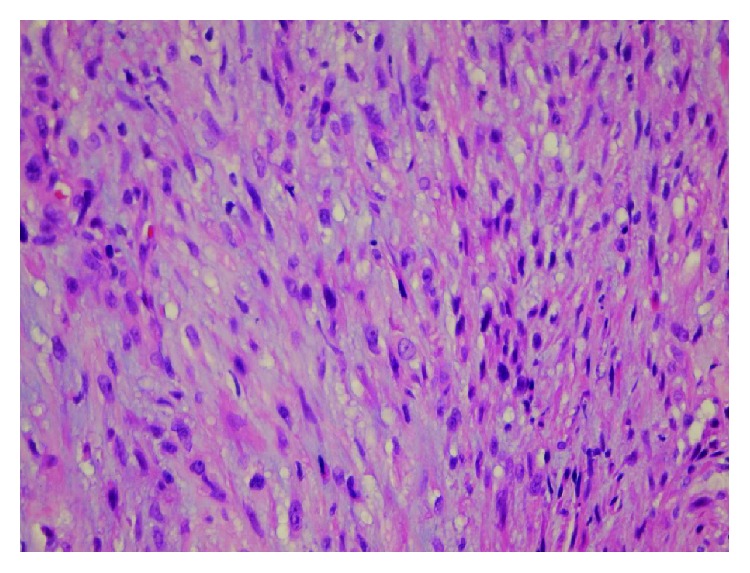
The histological section shows atypical tumor cells with fasciculated and interlacing pattern. H&E 40x.

A 58-year-old woman was admitted to a cardiovascular surgery clinic for the evaluation of a painless mass in her left leg and lower extremity edema with a three-week duration of history in August 2008. Physical examination showed a mass was palpable deep to the skin in the course of the short saphenous vein. No other skin lesions and no systemic manifestations were found. Routine laboratory studies were unremarkable. Ultrasonography detected a luminal mass in the short saphenous vein. A magnetic resonance imaging (MRI) revealed a 15 × 10 × 7 mm heterogeneous mass extending into the Achilles tendon, which may be related to the thrombosis of the short saphenous vein. The mass was excised en bloc under tourniquet control with a safe margin. Vascular reconstruction was not performed. The gross specimen consisted of the saphenous vein was surrounded and infiltrated by a whitish to brownish, rubbery tissue all along its course, measuring 3.5 × 2 × 1.5 cm. Microscopic examination showed a tumor was arising from the vascular wall (Figure 1) and composed of spindle-shaped cells, arranged in intersecting fascicles, with fusiform nuclei displaying moderate atypia with hyperchromasia, nuclear enlargement, and occasional giant cells (Figure 2). Mitotic count varied between 7 and 8 per 10 high-power fields, with occasional atypical mitotic figures. Immunohistochemically, the tumor showed strong and diffuse reactivity for smooth muscle actin and focal and moderate reactivity for desmin and caldesmon. The proliferation index detected by Ki-67 was found to be 20%. The immunostains for S-100 and CD34 were negative. At the time of diagnosis, the patient has no metastasis. Postoperatively, the wound healed well with normal extremity function. Although the patient was referred to an oncologist to plan the follow-up strategy but, she denied. The patient was medically well in 5 years, but at the end of the five-year period, she was readmitted with local recurrence. After the reoperation, the patient received adjuvant radiotherapy and chemotherapy. She underwent regular controls at the Department of Oncology, she remains alive and, in her last following period, there was no evidence of metastasis or local recurrence.

## 3. Discussion

Leiomyosarcomas of soft tissue generally present retroperitoneum; however, it is the predominant sarcoma arising from blood vessels. vLMS more commonly arises from the venous, rather than arterial vessels. It is reported that the long saphenous vein is the most affected vein and followed by the femoral vein and popliteal vein in the lower extremity [3]. On the other hand, there are case series pointing out that the majority of tumors were from the femoral vein [4, 5]. This is, to our knowledge, the second case of primary vLMS of the short saphenous vein. The first one was published in 1986 by Leu and Makek [2].

The symptoms are related to the location of the tumor, rate of tumor growth, and degree of collateral blood flow. They most commonly present as slow-growing, painless masses. When the tumor is occlusive, the symptoms can mimic those of deep vein thrombosis [6, 7]. In addition, in the initial radiologic evaluation, the tumor is often misdiagnosed as a deep vein thrombosis, similar to what occurred in our case. Abed et al. reported that most patients had been thought to have deep venous thrombosis resulting in diagnostic delay [4]. Cross-sectional imaging with contrast-enhanced CT or MRI is recommended in differentiating intravascular tumor growth from thrombosis; however, findings may be nonspecific [8, 9].

Histological tumor was composed of spindle-shaped cells with an eosinophilic cytoplasm with muscular striation and cigar-shaped rounded nuclei. We applied immunohistochemical staining for smooth muscle markers such as actin and desmin as well as h-caldesmon to verify the diagnosis. The differential diagnosis includes spindle cell-shaped neoplasms such as benign and malignant tumors of the nerve sheaths, myofibroblastic tumors (myofibromatosis, fibromatosis, and myofibroblastic sarcoma), synovial sarcoma, and fibrosarcoma.

Leiomyosarcomas have clinical and biologic differences according to site-related subgroups. Although leiomyosarcomas of the retroperitoneum and abdominal cavity are associated with an aggressive clinical course, the behavior of the primary vLMS has been controversial. Leu and Makek reported that small vLMSs might have relatively good prognosis [2]; however, Berlin et al. reported metastases, even with relatively low mitotic rates of tumor [10]. Italiano et al. determined that vLMS had a significantly worse median metastasis-free survival and an overall survival than leiomyosarcomas of other origins [11]. The more aggressive behavior and worse prognosis of vLMSs could be due to a direct attack to the vascular system [1]. However, in another study comparing the prognosis of vLMS with leiomyosarcoma other anatomic sites, authors reported that vLMS of extremity has similar prognosis to leiomyosarcoma of different origins [12].

Operation is the mainstay of treatment for vLMSs. The role of neo-adjuvant chemotherapy (CT) and radiotherapy (RT) in the management of disease is still unknown. The rate of local recurrence was not significantly different between patients who received perioperative RT and those who did not [13]. Similarly, Italiano et al. showed that RT has only a limited role in the management of vLMSs and they founded that 75% of patients died of metastatic disease within the first 3 years of diagnosis, although good local control by surgery and RT was achieved [11]. CT was mostly offered to treat metastasis; however, its advantages have not been well documented [14]. The authors reported that neither systemic CT nor chemoembolization or radiofrequency has improved the overall survival or disease-free survival [14, 15].

Although there has been local recurrence in the five-year period, the present case seems to be a good prognosis. The review revealed that the survival of the deceased patients with the vLMS of the popliteal artery was between 7 months and 4 years [16]. Reix et al. described a series of six patients with a leiomyosarcoma and one with hemangioendothelioma. In their series, the median survival was reported as 31 months [5]. Marle et al. reported that the average survival was 4 years (range 1 month to 17 years) [17]. In those studies, the majority of patients with vLMS have metastatic disease at the time of resection. In the present case, the patient was still alive 60 months following reoperation of recurrent tumor. We thought that this finding may be associated with the localized disease of our patient.

In conclusion, vLMSs of extremity are rare and aggressive tumors. Clinicians should consider that it may be misdiagnosed as deep vein thrombosis in the initial diagnosis. The management of these tumors is surgical treatment; however, considering the local recurrence in our patient, radiation therapy and chemotherapy should have been considered. Due to its rare incidence, all publications in the literature are of small clinical series or case reports. Therefore, we thought that our case report also provides data to the literature for the management of vLMS.
